# The Present Etiological Status

**Published:** 1883-06

**Authors:** 


### Editorial.

### THE PRESENT ETIOLOGICAL STATUS.

In the first number of the present volume of the INDEPENDENT PRACTITIONER, we said: "At the present time the tendency in investigation is largely toward the etiology of dental diseases, and in this field we hope that this journal will do some effective work." No one will deny that this implied pledge has been quite fulfilled in the six numbers already issued. Five valuable papers have been presented, and no dental journal can boast of having done more to add to the knowledge of the origin and character of the diseases which dentists combat than can the INDEPENDENT PRACTITIONER. Other valuable contributions are yet waiting, which will be published in due time. Yet we are not sanguine of soon seeing this much vexed question definitely settled for all time.

It is quite enough if we can be the medium through which additional light may be obtained, and a better understanding of the laws which govern oral diseases be gained. The advocates of the several theories are all tenacious in their beliefs, and each is assured that he alone possesses the true key to the mysteries of oral pathology. It is well that this is so, for a half-hearted advocate will scarce make any decisive progress. The world likes positive men. Yet one should not be so dogmatic as to ignore unpleasant truths. No single hypothesis has yet been presented which is unassailable. There are so many factors which may possibly make up the sum of the etiology of caries that the creed of Dr. Miller would seem to commend itself to the judicial and unprejudiced mind, when he says: "Fungi, even when reinforced by acids, do not in all cases prove themselves sufficient for tooth disorganization. There appears, in many cases at least, to be some other element at work."

The man who is already committed to a theory will not, perhaps, be enabled to subscribe to this, yet we are certain that if the enthusiastic advocates of the various exclusive hypotheses were fully acquainted with the views held by the others, there might not be such irreconcilable differences as at first sight would be suspected.

The large attendance at the Springfield meeting was mainly due to the fact that it was generally understood that the subject of etiology would be thoroughly considered, and it was amusing to see how entirely the "bacterians" were misunderstood. Perhaps this was due in part to the injudicious presentment of that hypothesis by some of its zealous advocates, who themselves are not very clear in their conception of the theory. They were represented as teaching that micro-organisms are living, breathing, sentient, malignant beings, that of malice aforethought attack and actually devour sound, healthy teeth. Any one who refers to these organisms as "bugs" only advertises his own ignorance. We are not an advocate of the purely bactarian origin of tooth decay, but we do not like to see a theory condemned upon false grounds.

As we understand the teachings of that school, they simply affirm that if the vitality of a tooth be sufficiently lowered and its structure be favorable, micro-organisms—vegetable fungi—may enter it and induce the destructive kind of fermentation which takes place in all organic, devitalized matter under favorable conditions, and that the organic portion once destroyed, the inorganic soon crumbles, and thus the tooth is demolished.

The advocates of the purely chemical theory, on the other hand, ignore any part which the organic or living portion of the tooth may play in caries, and impute it to a simple chemical solution of the inorganic matter.

Those who hold to the inflammation hypothesis believe that some outside influence initiates the pathological condition, which then pursues a course somewhat analagous to that in ordinary tissues.

The observations of Dr. Miller lead him to the conclusion that the first step in dental caries is from without, and is brought about through the action of some acid, and that the action of this acid precedes the invasion of fungi which, the path once opened for them, may play an important part in tooth demolition. He believes also that there are other factors, not yet fully understood, which are active in this destructive metamorphosis.

It may be seen that, correctly understood and interpreted, all these hypotheses may not be entirely inharmonious. Even the bacterians admit that some agency precedes the invasion of micro-organisms, since they acknowledge that caries presupposes a certain pathological condition. All the others agree that this primary action is doubtless due to acids. The area of softened dentine of Miller, and the zone of inflammation of Abbott, may be identical. But the observers interpret certain appearances differently, and so, after all, the main points of divergence are in the various readings of the microscope, and thus the apostles of the separate theories are not so far asunder as might at a glance be supposed.

Concerning the great good which has resulted from the studies of all these observers there can be no question. To Drs. Bodecker and Abbott, to Prof. Mayr and Dr. Stockwell, and last, but not least, to the exhaustive researches of Dr. Miller, the profession owes a debt of gratitude which it will in time fully acknowledge. The end is not yet, for the last named of these gentlemen has but just entered upon an entirely new series of observations which, we doubt not, will throw yet more light upon this hitherto dark subject.

It may not be unprofitable to very briefly review the opinions held by different writers upon dental pathology.

The earliest authors presented no definite theory, but Fox believed that caries originated within the tooth. He called it "inflammation in the bone of the tooth, resulting in mortification."

William Robertson, in 1835, first propounded the purely chemical theory.

John Tomes, in 1848, called caries a "death of the tissue and loss of the power of resistance to the action of acids."

Charles Tomes leans more toward the views of Robertson, although he does not ignore the vital influence.

Magitot supports the Chemico-vital theory of John Tomes.

Wedl takes substantially the same view.

Bridgeman, (English prize essay upon dental caries) attributes decay to purely electrical conditions.

Spence Bate, of Plymouth, England, lays it to the existence of carbonic acid in abnormal quantities.

Leber and Rottenstein to Leptothrix, or cryptogamous growths. With the views of later writers our readers are doubtless well acquainted.

